# The experience of gay Christian men attending collaborative workshops facilitated by a sexual health professional and a priest

**DOI:** 10.1093/sexmed/qfaf103

**Published:** 2025-12-17

**Authors:** Remziye Kunelaki, Zoe Bennett, Selim Cellek, Carrie Roder

**Affiliations:** School of Allied Health and Social Care, Faculty of Health, Medicine and Social Care, Anglia Ruskin University, Chelmsford CM1 1SQ, Essex, United Kingdom; Cambridge Theological Federation, Westminster College, Cambridge CB3 0BJ, United Kingdom; Medical Technology Research Centre, Anglia Ruskin University, Chelmsford CM1 1SQ, Essex, United Kingdom; School of Medicine, Faculty of Health, Medicine and Social Care, Anglia Ruskin University, Chelmsford CM1 1SQ, Essex, United Kingdom

**Keywords:** gay, Christian men, sexual health, workshops, collaboration, psychosexual therapist, priest

## Abstract

**Background:**

Although workshops facilitated by a sexual health professional and a priest to support gay Christian men who struggle with accepting their sexuality and religious identity have been organized, the experience of attending such workshops has not been studied.

**Aim:**

To understand the experience of gay Christian men who attended collaborative workshops.

**Methods:**

Six collaborative workshops were conducted between a sexual health clinic and a church in central London from January to September 2018. Purposive sampling was used to recruit 11 gay Christian men who participated in the workshops. Data on their experiences were collected through semistructured interviews, images, and the innovative method of haiku poetry, which was used to reflect on their experiences during and after the workshops. All the data were analyzed using thematic analysis.

**Outcome:**

Gaining insights into the experiences of gay Christian men who participated in collaborative workshops.

**Results:**

The collaborative workshops had a significant impact on gay Christian men in two key areas: self-perception and relationships. The contributing factors to this influence were either environmental or personal. Three themes emerged: motivation, self-development, and the influence of the environment. Some participants embraced the workshops, while others experienced a sense of urgency. The workshops were experienced as fostering their self-growth. In contrast, others were reminded of their destructive behaviors, which left them feeling hopeless. For some participants, the workshop’s environment highlighted their minority status as a strength, whereas for others, it highlighted their loneliness.

**Clinical Implication:**

This study showed the importance of understanding motivation for attending the collaborative workshops.

**Strengths and Limitations:**

Although the findings cannot be generalized to all religious and sexual minorities, they can inform future collaborations. Although a small sample was recruited, it provided an in-depth understanding of the experiences of these men. Limited funding restricted the workshops’ accessibility to hard-to-reach communities. The data were analyzed from a psychological perspective.

**Conclusion:**

This study introduces a novel model for integrating religion and sexuality. It adapts Maslow’s hierarchy of needs for gay Christian men to include freedom from shame as an essential requirement, subsequently fostering belongingness, connection, and love.

## Introduction

Some gay religious men, such as Christian gay men, often grapple with dual identities. Sexual identity encompasses the personally chosen labels that individuals adopt to express their understanding of their sexuality.^1^ Religious identity refers to an affiliation with a particular religious institution, typically signifying some level of spiritual and communal involvement.^2^ It remains uncertain whether religion serves as a risk or a protective factor for LGBT+ communities.^3^ Religion or spirituality can act as a risk factor when sexual minorities engage in religious or spiritual practices that condemn their sexuality, resulting in emotional distress.^4^ Tan discovered that high levels of spiritual well-being and nurturing a positive relationship with spirituality among gay men serve as an adjustment factor, contributing to greater self-acceptance.^5^

The idea behind this research was born through the caseload of a psychosexual therapist practicing in the National Health Service (NHS) in a busy sexual health and human immunodeficiency virus (HIV) department in London. Patients who identified as gay and religious, no matter the psychosexual presentation to which they were referred, were observed to be struggling with the two identities. At that point, a collaboration between the psychosexual therapist and the priest of the nearest church (Church of England) to the clinic was trialed, involving church members and patients from the caseloads of psychosexual therapists.

The patients who attended therapy were unable to make sense of their polarized identity, which caused them issues with their mental health, and this often manifested in a lack of sexual well-being. There was a struggle to be accepted in both worlds, the gay scene and the Church. Lesbian, gay, bisexual and trans (LGBT) young adults who mature in religious contexts have a greater chance of experiencing suicidal thoughts and, more specifically, chronic suicidal thoughts, as well as suicide attempts, compared to other LGBT young adults.^6^

In the literature, gay Christian men raised in more conservative traditions, such as Evangelicals or Catholics, struggled more with their identities, as they believed that their sexual orientation meant they were sinners and would go to hell.^7^ Another qualitative study showed that gay Christian men felt misinterpreted and misrecognized as the stereotypical images about them were negative and deprived of opportunities; they were subjected to various degrees of harassment and exclusion from intimate relationships.^8^ In addition, Subhi et al. (2011)^9^ found that participants who experienced conflict between Christianity and homosexuality described personal effects such as depression, guilt, anxiety, suicidal ideation, and alienation.

After receiving anecdotal evidence of a successful, highly valued pilot collaboration, we aimed to explore further the experiences of gay religious men who attended those workshops. To ensure sample homogeneity, we concentrated on gay Christians.

Our goal was to understand what it is like to be a gay Christian man attending a series of collaborative workshops facilitated by a sexual health professional and the Church. The research question of the study was: “What is the experience of gay Christian men attending collaborative workshops facilitated by a sexual health professional and the Church?” There were two subquestions to the research question: (1) How did the workshops influence the gay Christian men? (2) What were the factors associated with their experience?

This study, with its findings, aimed to inform recommendations for both the Church of England and the sexual health and HIV departments within the National Health Service on ways to support religious and sexual minorities.

## Methods

The worldview of this study is rooted in the socially constructed paradigm of interpretive research. The study’s theoretical framework was interpretive. The intention was to describe more than just the experience reported at the end of the workshops. We aimed to interpret every aspect of the participant’s experience, considering who they are (based on my knowledge and experience of them), the potential impact of the workshop environment, and maintaining reflexivity in my position. In line with hermeneutic understanding, truth is how things are.^10^

The participants were invited to six consecutive workshops at the church from January to September 2018. Each workshop focused on a specific topic and was organized based on the attendees’ requests during the open evenings. The PowerPoint presentations by the sexual health professional and the priest addressed current evidence from psychological and theological perspectives. Each workshop featured 20-minute PowerPoint presentations, up to 60 minutes of group discussion, a short break, and 15 minutes for prayer and completing evaluation forms. After each workshop, participants were asked to provide feedback through haiku poetry. During the first workshop, they received a brief training session on writing haiku for feedback.

The six workshop themes included being a minority within a minority, feelings of shame and guilt, religion and sexuality, the coming-out process, sexual interactions among gay Christian men, and authenticity and integration. The priest and the sexual health professional shared a philosophy of inclusion regarding sexuality and religion. They believed that one can be gay and Christian in a way that is right for the individual.

The study participants were recruited through open evenings organized at the church, with promotions via social media, LGBTQ+ community magazines, charities, and sexual health clinics. Recruitment occurred during these open evenings using purposive sampling based^11^ on established inclusion and exclusion criteria. The inclusion criteria comprised men (including trans men) aged 18 and over, men who identify as gay, gay men who identify as Christian, and gay Christian men who grapple with the conflict between sexuality and religion and are willing to commit to attending all 6 workshops. All the participants were fully informed in writing and provided their consent to participate in the study, which received approval from the NHS Research Ethics Committee (17/EM/0448) and the Anglia Ruskin University Faculty Research Ethics Panel (FMSFREP/17/18/084).

Once the series of workshops concluded, participants were invited to a one-on-one interview, during which they were encouraged to bring images (photos, drawings, sketches, paintings) that best represented their experiences during the workshops. Semistructured one-on-one interviews were recorded and transcribed using secure digital devices, and the data were analyzed anonymously. These semistructured interviews were conducted in the privacy of the counselling room at the sexual health clinic; audio recordings were uploaded to an encrypted device, and hard copies were securely disposed of. The thematic analysis explored haiku poetry, textual imagery, and transcriptions.^12^ The recordings (including images and interviews) were listened to and transcribed using software called Google Docs. Each theme was linked to relevant data extracts. Codes were then grouped into subthemes and subsequent themes, which were checked against and referenced back to the original data. There was reflective note-taking throughout the research process for the researcher. Direct quotes were provided to demonstrate the analytic process. The analysis was structured around the data, with the primary aim of answering the research question.

A qualitative methodology was employed to understand the experiences of individuals who attended the collaboration without any preconceived agenda. This approach enabled the data to reveal the essence of these experiences. To grasp the depth of participants’ experiences, various methods were employed to capture their feelings about attending the workshops immediately after the workshops (through haiku poetry) and upon completion (through images and semistructured interviews).

Utilizing haiku poetry to document immediate experiences enhances the study’s innovative nature. The poems were assessed for clarity of handwriting during the interviews. Haiku poetry analysis was conducted line by line, ensuring the capture of experiences that could vary across multiple themes in a poem. The poems were printed, and notes were made on the side to begin the formal coding process. As haiku poetry involves 17 syllables at a time; the codes were either linked to words used by participants or explored and identified a more profound meaning. The identified codes were grouped into subthemes and later into themes.

Textual analysis of the images relied heavily on participants’ spoken words. The words used to describe the images were analyzed using thematic analysis. The steps of thematic analysis were followed to identify codes, capturing what was said and the corresponding image for each participant. The codes were then grouped into themes.

The transcript analysis of the interviews was conducted using a thematic analysis approach. The researcher transcribed each interview to immerse themselves in the data. Each transcribed interview was read and re-read several times, while taking initial notes and keeping a reflective journal. The re-read notes were given a descriptive name, such as a container called “code.” NVivo, a software program, helped to link each section of the transcripts with a code. The codes and terms in NVivo were defined and listed directly within the transcript. 15 – Checkpoints^13^ were applied to the workshop data’s thematic analysis.

## Results

The sample of participants consisted of 11 men. They all identified as gay and Christian, aged between 27 and 73 years old. Five men were identified as White British, 2 as Black British, 1 as Chinese, 1 as White Other, 1 as White Irish, and 1 as having an Any Other Asian Background. In terms of denomination, 6 men identified with the Church of England, 3 with the Catholic Church, 1 with the Baptist/Pentecostal tradition, and 1 with the Charismatic tradition. Three men were in long-term relationships at the point of the interviews.

The experiences of gay men who identified with conservative churches, such as Roman Catholics or Pentecostals, revealed a deeper struggle with their sexual orientation than those affiliated with more progressive churches, as shown by both literature and data. The study’s physical location contributed to a sense of safety and personal development. However, the inclusivity described by some participants was not universally felt within the Church of England. Participants from more conservative denominations expressed feelings of *hopelessness*, with their motivation to attend the workshops stemming from a strong desire for acceptance and a sense of belonging. This study does not seek to explore the differences between conservative and progressive churches; instead, it focuses solely on the Church of England.

### Analysis of semistructured interview transcripts

The analysis of the interview transcripts revealed three themes: *motivation*, *self-development*, and the *influence of the environment* (Table 2). *Motivation* reflects the participants’ mindset before attending the collaboration and highlights their goals. “The first step was fundamentally about who I see myself as and how I envision my faith and sexuality coexisting; it was almost like building blocks for this new stage in my life” (P018). Some participants embraced the workshops’ experiences and were open to their potential benefits. “I thought I was going to have a revelation moment, realizing how everything makes sense” (P014). In contrast, others had limited expectations for the workshops, seeking specific answers, which created a sense of *urgency* in their experience. “Right now, I am sort of struggling with it (conflict of identity) because the church has hurt me so much” (P020).

*Self-development*, as a theme, summarized the stages participants experienced in their self-reflection. Some participants found the workshops enhanced their *self-growth*: “Even if the workshop were finished, you would reflect after each session; you are still thinking” (P012). The workshops were experienced in a way complementary to the stage of some participants’ self-growth. “Most of the things I got out of the workshop did not necessarily come from speaking; I did a lot of the internal processing, which helped to a greater extent” (P014). Some participants found the workshops’ input aligned with their process, which enhanced their self-growth: “During the collaboration, I found a partner, and I think it (the collaboration) has done a lot to push me through the journey of self-acceptance and enjoyment” (P018). Conversely, some participants, through the workshops, were reminded of their destructive behaviors. Their sense of self-development was halted due to their substantial experience of destructive behaviours, which left them feeling *hopeless.* “I discovered sex through basements, but I know that is unhealthy… I had to be in the basement that night (after a workshop)” (P016).

The third theme of the workshops, *the influence of the environment*, encompassed the subthemes *being a minority within a minority*, *physical location*, *group dynamics*, and *safety.* The workshop’s environment highlighted for some participants their multiple minority statuses, such as being gay, Christian, or a foreigner. “I was delighted to be part of the group—the breadth of shared experience, in terms of age and ethnicity” (P017). For some participants, *minority* status was linked to “strength,” while for others, it represented “loneliness”: “I sometimes left feeling exhausted after the groups from what I was hearing: I thought these people were miserable, so when you left on the main street, you had just heard these very precious things people were sharing about faith, and suddenly you were back out on the street again” (P016). The *physical location* emerged as a significant subtheme, where the “setting” of the workshops played a crucial role in fostering a sense of “community” and emphasizing the importance of “inclusive churches”: “I am here, I am in the place, in the capital of all; you find yourself pinching yourself; this is where it all happens…” (P013). *Group dynamics* formed another subtheme that captured the *human interactions* within the workshops’ environment: “Some of the group members treated the workshop as their therapy session. People would recount the same things, or revisit a personal anecdote they had already shared, and we had already engaged with, but they kept playing the same record” (P014). Finally, some participants experienced a sense of *safety* in the workshop environment: “I loved it. I loved it. I love that I felt very comfortable: it was a fantastic experience and seemed welcoming and safe. Moreover, because people were sharing their stories, it felt very secure. It was gentle. It was lovely” (P016).

### Haiku poetry analysis

The analysis of haiku poetry (Table 1) revealed two main themes: *possibilities* and the *environment* of the workshops.

**Table 1:** The haiku poetry analysis. Two main themes, possibilities and environment, were identified from the haiku poetry written by the participants. The subthemes describe a concept underneath the more extensive umbrella of a theme. The codes describe the process of identifying the data with a concept and identifying a relation between the extracts. The “extracts” column gives examples of haiku poetry written by the participants. The participants are given an anonymized number starting with “P.”

**Themes**	**Sub-themes**	**Codes**	**Extracts**
**Possibilities**	*Positive outlook*	“Light at the end”	“Fruit, nuts, seeds will come” P013
		“Ongoing healing”	“To heal the scabs” P011
	*Hopelessness*	“Ongoing shame”	“Hiding to survive” P012
		“Ongoing regret”	“They don’t know me, it’s too late” P016
		“Yearning for intimacy”	“Yearning to find another to commune with” P017
**The environment of the workshops**		“trusting”	“As the guard comes down” P014
	*Safety*	“held”	“More gently here” P017
		“isolation”	“Exiled again” P010
		“revelation”	“Apocalypse now” P013
		“courage”	“Do not hide your life” P018
		“acceptance”	“Being myself at last” P012
	*Preparedness for integration*	“intimacy”	“Consenting adults with intimacy” P011
		“connection”	“Now a bridge spans the valley” P014
		“authenticity”	“Transparently authentic” P010
		“hiding”	“I am afraid above ground” P016

**Table 2:** The analysis of semistructured interviews. Three themes were identified from the interview: motivation, self-development, and the influence of the environment. The subthemes describe the different patterns across the data. The codes are categories to describe the data, and the extracts are direct quotes from the participants.

**Themes**	**Sub-themes**	**Codes**	**Extracts**
**Motivation**	*Receptivity to messages from the workshops*	“preparation”	“It was like building blocks this new stage in life” P018
		“coming out”	“I thought if I ever met someone, that person will never meet my family” P016
		“Peer support”	“What is good about this workshop, you meet other people” P012
		“revelation”	“This revelation moment thinking of that how everything makes sense” P014
		“partnership”	“Being single and potentially finding a new partner” P011
		“learning”	“You never stop learning” P010
		“No expectation”	“I thought I would go along and see what it is about” P017
	*Urgency to be validated and accepted*	“shame”	“Because I had problems with shame about sexuality” P015
		“discomfort”	“I was uncomfortable with my sexuality” P019
		“struggle”	“I am struggling because the church has so hurt me “P020
**Self -development**	*Self-growth*	“Willingness to be open”	“I stopped in the first room—where you can pray” P012
		“self-reflect”	“I did a lot of the internal processing” P014
		“learn”	“I felt I learned a lot about sexual health “P019
		“Permission giving”	“When the Rev talked about masturbation” P014
		“whole”	“It is about the whole person, the human” P016
	*Hopelessness*	“Basement sex”	“I had to be at the basement that night” P016
		“Pessimism about coming out”	“I do not see myself coming out” P019
		“regret”	“By not being out, I feel I am betraying people” P012
**The influence of the environment**	*Being a minority within a minority*	“Age, ethnicity, and denomination diversity”	“The breath of shared experience, age-wise and ethnicity” P017
		‘Strength in diversity”	“Whether young or old, whoever you are, you have a place, an opportunity to love and be loved” P010
		“loneliness”	“You heard these very precious stuff people sharing about their faith, and suddenly you are out in the street again” P016
	*Physical position*	“The location”	“I am here, I am in the place, in the capital of all where it happens” P013
		“community”	“I felt part of the community very much” P019
		“Comparison to less inclusive churches”	“The other churches would not dream of ever talking about sex in a place of worship” P014
	*Human dynamics*	“Individual process”	“When I was coming to this group, I felt part of this group” P012
		“The combination of co-facilitators”	“Seeing you was wonderful; you make an excellent team” P010
		“Holding back”	“Feelings of entertaining suicide I was curious if everyone in the room had [at] some point considered it” P013
		“frustration”	“I thought you need to ‘shut up’” P015
		“disengagement”	“I felt this was not helping me, and I was disengaged” P014
	*Safety*	“innovation”	“The whole thing was cool” P018
		“Trust in science”	“Your presentation is one of the things I loved about you” P010
		“Jesus’s approval”	“He (Jesus) will be in the middle of it” P016
		“Integration and authenticity”	“The last one (workshop)when everyone was in the same mindset and that felt more emotional for me” P012
		“disclosure”	“I mentioned in the first meeting that I was a priest and did not tell people as much” P016

**Table 3:** Overarching themes.

**Themes**	**Subthemes**
**The environmental factors**	*Physical position*
	*Human dynamics*
	*Sense of community*
	*Being a minority within minority*
**The personal factors**	*Receptivity to messages of workshops*
	*Urgency for validation and acceptance*
**The influence of self-perception**	*Positive outlook*
	*Hopelessness*
	*Self-growth*
	*Integration*
**The influence on relationships**	*Validation*
	*Connection*
	*Safety*
	*Nourishment*

*Possibilities* surrounded the stage where participants understood their paths beyond the workshops. For some, the experience held a *positive outlook*; for others, it felt *hopeless.*

The workshop environment influenced the experiences of the gay Christian men who participated, fostering a sense of s*afety* and *preparedness for integration*.

The experiences of the participants expressed in their haiku poetry can be summarized in three groups:

1. They were reflective and traced the integration of their religion as the workshops were co-delivered.

2. The collaboration highlighted the ongoing shame and guilt because of being gay and Christian, but the experience was one of appreciation and hopefulness.

3. The collaboration environment had a significant impact on the experience of some participants.

### Imagery analysis

**Figure 1:**
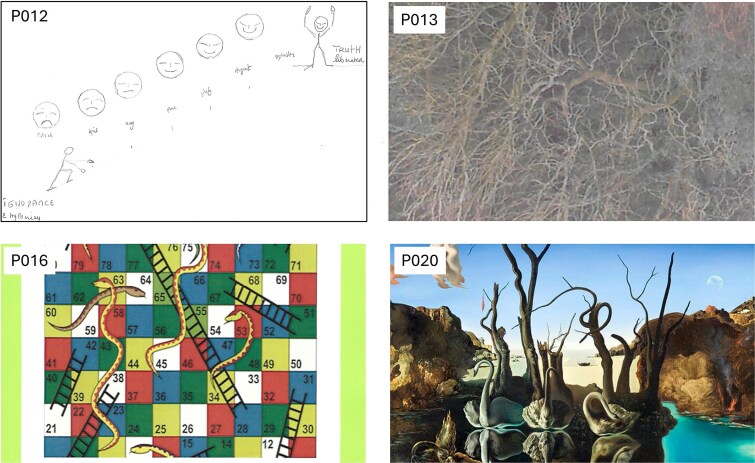
Four images selected by the participants (P012, P013, P016, and P020) were within the theme of being validated.

**Figure 2:**
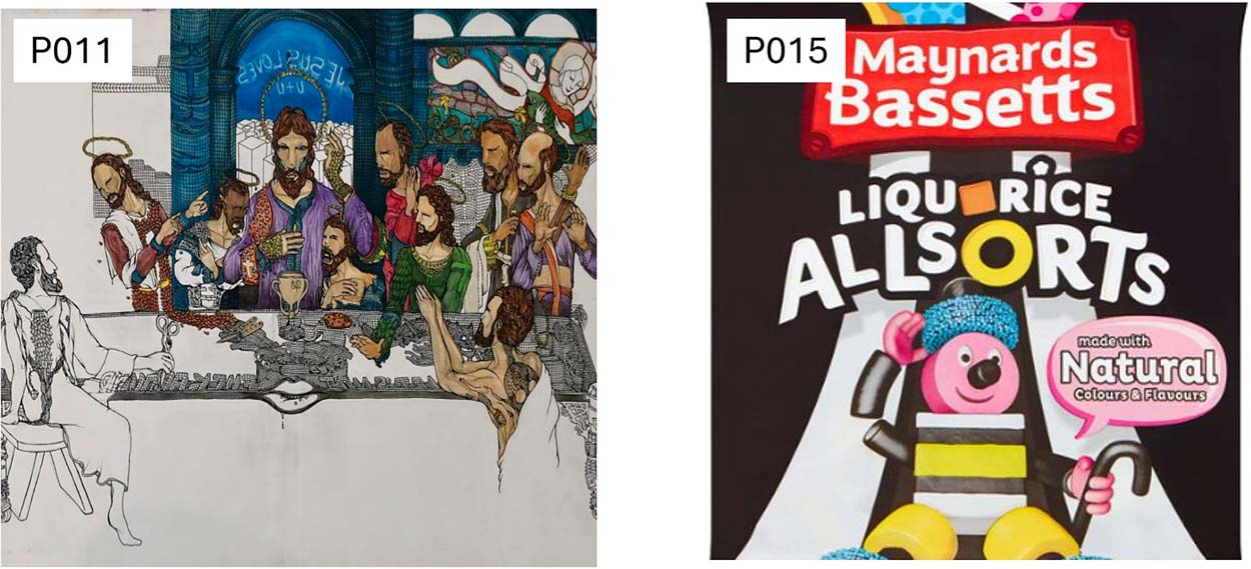
Three images selected by the participants (P010, P011, and P015) were within the theme of connection. Participant P010 did not consent to the image he selected to be included in the publication.

**Figure 3:**
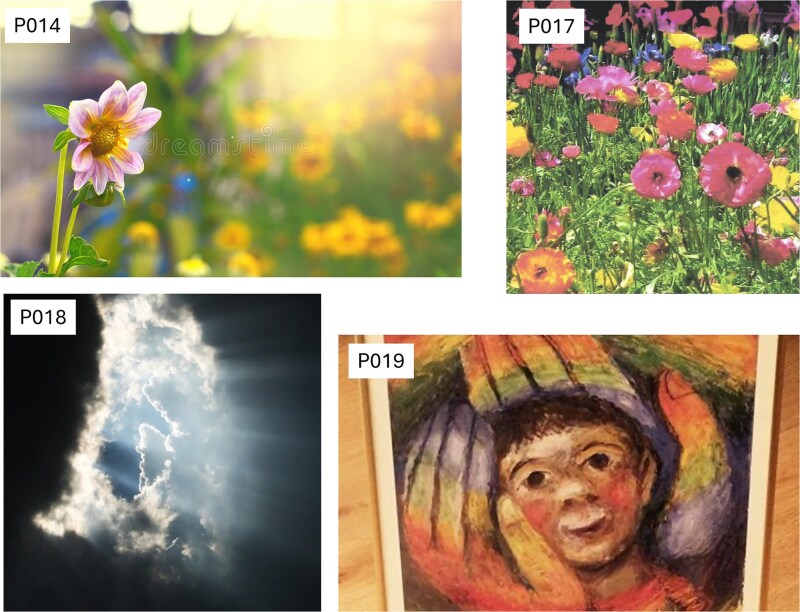
Four images (P014, P017, P018, and P019) were selected by the participants and related to the theme of being nourished.

The main themes identified in the textual imagery analysis were experiences of being *validated*, *connected*, and *nourished*. The imagery was the only data collection method that allowed participants time to reflect on their experience attending the collaboration. The theme of feeling *validated* was vividly captured in the images. These images (Figure 1), including a drawing of a journey to truth (P012), a photo of a naked tree in winter (P013), a board game of snakes and ladders (P016), and a painting by Salvador Dali (P020), depicted the participants’ experiences of recognition and acceptance as gay Christians. The theme of *connection* (Figure 2), symbolized by the Eucharistic table (P011), reflected the participants’ experiences of coming together. Other images that captured this theme were a photo of couples lined up (P010—no permission to share) and a packet of sweets named All Sorts (P015). The theme of being *nourished* signified the experience of being looked after in a way that was right for the participants. The images associated with the theme of being *nourished* (Figure 3) were an image of a flower looking at the sun (P014), a picture of a meadow of flowers (P017), a photo of the sky clearing from the clouds (P018), and a painting of a head being held in two hands (P019).

### Overarching themes

Once the three methods were analyzed, the overarching themes and subthemes were examined (Table 3). The three methods captured participants’ experiences at different stages of their attendance at the collaboration. The haiku poetry showed the participants’ experiences after each workshop and helped tell the story of their journey as the workshops progressed. The images captured the participants’ reflections in their own time after the series of 6 workshops concluded, and the interviews provided an opportunity to discuss their experiences in the privacy of a consulting room.

## Discussion

This study aimed to understand the experiences of gay Christian men participating in a collaboration led by a sexual health professional and a Church of England priest. The results indicated that attending these workshops had a positive impact on the self-perceptions and relationships of some participants. Some participants reported that the workshops supported their personal growth, attributing this to the new insights gained and the opportunity to reflect on their identities as gay and Christian men. This aligns with the findings of Lease et al.,^14^ which established that positive or affirming group faith experiences correlate with reduced internalized homonegativity and improved spiritual and psychological well-being for LGB individuals. The current study captured the experiences of a more diverse population, extending beyond the exclusively white participants of Lease et al.^14^

Its epistemological position is socially constructed, reflecting the accurate experiences of gay Christian men who attended the workshops at the moment they occurred. Ontologically, this study leans toward the relativist end of the reality spectrum. It employs a qualitative methodology using the phenomenological approach to distill the essence of human experience.

One of the most common themes among religious lesbian and gay clients who seek therapy from Christian counsellors is achieving harmony between the two identities.^15^ The current study similarly demonstrated that the personal growth experienced by some participants was attributable to the example set by the psychologist and the priest, who also expressed an affirmative stance toward accepting one’s sexuality and religion.

The workshops provided a confidential space for participants to explore integrating their sexuality and religion. Similar to the study by Rodriguez and Ouellette,^16^ participants who experienced the most significant integration were those who were open or became open about their identity outside the workshops.

Some participants attended with a sense of urgency to be validated and accepted for their sexuality and religion, coupled with hopelessness in achieving that acceptance. The hopelessness regarding the integration of these two identities experienced by some participants was linked to pre-existing issues such as shame and regret for not having come out. Participants who identified with conservative denominations, such as Roman Catholics, experienced the most incredible hopelessness regarding the integration of their sexuality and religion. This finding aligns with existing literature.^17^^,^^18^

Some participants who had not reconciled with their multilayered identities found the collaboration inadequate; this was particularly true for those who had not yet come out as gay to their families or communities. The fear of rejection can lead to an existential crisis for sexual minorities who adhere to specific doctrines throughout their lives, only to be told that they are sinful in moments of need and vulnerability.^19^

Some participants restricted their self-expression due to frustrations with interpersonal dynamics during the workshops or a lack of assertiveness. A few participants became disengaged during the sessions or felt a strong urge to disconnect immediately after they ended. They observed others dominating conversations in the workshops, while some hesitated to address specific topics. Subjects that were not tackled during the workshops included the effects of pornography and suicidal ideation on gay Christian men. Evidence suggests that LGB individuals often conceal or repress aspects of their identity in social interactions, presenting as “selectively inauthentic.”^20^ Participants felt isolated, perceiving themselves as distinct from others in the group. Given the age diversity, some participants shared experiences of facing discrimination, oppression, and the loss of loved ones to HIV, all of which are complex factors linked to internalized homonegativity among middle-aged and older gay and bisexual men.^21^

All the images presented in the interview schedule reflected the workshop environment. The atmosphere of the collaborative workshops profoundly influenced participants’ experiences. For more conservative denominations, these workshops represented their first opportunity to express their identity as both gay and Christian. The co-facilitators and the group encouraged the formation of a community where some participants could come out for the first time. The co-facilitators embodied both aspects of religion (the priest) and sexuality (the sexual health professional). Furthermore, the workshops were held on the Church grounds, located in a neighborhood central to the gay community, which was particularly significant.

**Figure 4:**
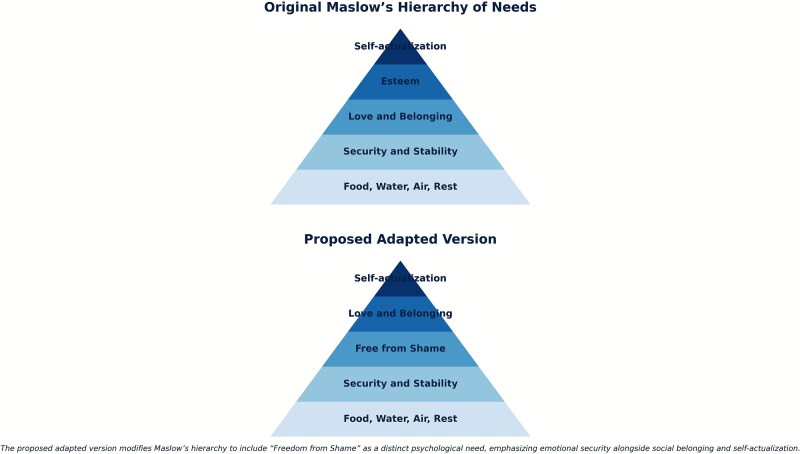
The original Maslow’s hierarchy of needs is at the top, and the proposed adapted version for the needs of gay Christian men is at the bottom.

One of the most significant contributions of this study was its consideration of the needs of gay Christian men. To this end, an adapted version of Maslow’s Hierarchy of Needs^22^ was proposed as a tool. Maslow’s pyramid has five levels of needs: physiological (food, water, warmth), safety (security), love and belonging (friendships), esteem, and self-actualization (Figure 4).

This research contributes to the proposal of an adapted structure of Maslow’s Hierarchy of Needs. The adapted version of this pyramid includes an additional level between safety and security and love and belonging. This new level is “free from shame.” “Free from shame” captures a new level of the pyramid where gay Christian men can let go of the shame for their sexual and religious identities. Cognitive dissonance^23^ arises when a person experiences tensions between two psychologically inconsistent thoughts or beliefs. The two identities, gay and Christian, were polarizing the participants existentially, leading to feelings of shame. The added level, “free from shame,” addresses the need to reconcile the cognitive dissonance in those who struggle with their sexual and religious identities.

Maltz^24^ wrote about the hierarchy of sexual interaction; sexual energy is channeled through two routes: the path of disintegration/disconnection or integration/connectedness. For the participants who experienced shame about their identity, their sexual energy was more likely to be channeled in the former route, which maintained their cognitive dissonance between their religion and sexuality.

Those participants who felt free from shame for being gay and Christian benefited the most from the workshops’ content and were able to relate it to their personal lives. This is a crucial level on the pyramid to move to healthy sexual behavior free from shame for being gay and Christian. For some men, being sexually active might not be relevant; however, for others, sexuality and the expression of it, free from shame, mattered. Whether one is sexually active or not, this added level in the pyramid will enhance the way they will experience the next level.

As a result, *belonging*, *connection*, and *love* were reflected in these participants’ experiences through the images, as they felt validated, connected, and nourished. Furthermore, a few of these participants experienced integration and authenticity at the end of the workshops, similar to self-actualization. This is in contrast to those who maintained high levels of shame about their sexuality, who were unable to connect with others in the group and their private lives.

While Maslow’s theory is secular, it acknowledges the human need for meaning, purpose, and transcendence, which are also central to many religious beliefs. Maslow (p. 382) described it as ‘[a] musician must make music, an artist must paint, a poet must write to be ultimately happy. What a man can be, must be'. *Self-actualization* and *authenticity* are terms used by some participants after the last workshop, titled “integration and authenticity,” where all the topics of the sessions came together, where they felt most at ease and connected.

Humans need interpersonal attachments and a sense of belonging to other people, which is considered fundamental to the species.^25^ Some participants who attended this collaboration were previously rejected and discriminated against for being gay and Christian. According to Baumeister and Leary,^25^ “social exclusion may well be the most common and important cause of anxiety.” For some participants, the need for belonging and love was crucial, and even the collaborative workshops could not provide that due to past experiences and the shame they carried. Foster et al.^26^ have previously studied the importance of safe spaces for sexual minorities to integrate sexual and faith identities and build spiritual resilience. In their study, contextual factors associated with integrating the two identities included having an accepting pastor who openly speaks about LGBT issues or finding other church members committed to LGBT social justice issues. Regular and sustained interaction with an accepting priest would likely help alleviate feelings of shame; however, the limited duration of these workshops did not allow for this for a minority of participants.

This is the first time Maslow’s hierarchy of needs theory has been used to discuss gay religious men. The findings suggest that religion played a crucial role in shaping the participants’ identities. The adapted version, as suggested by the findings, could offer a specific structure for considering the needs of gay Christian men.

### Limitations of the study

Although the findings cannot be generalized to all religious and sexual minorities, they illustrate the benefits of collaboration between sexual health and religious organizations. Recruitment proved challenging as attracting gay Christian men is complex, given that they represent a hard-to-reach community. Furthermore, local authorities faced restrictions when promoting the collaboration imposed by the local sexual health commissioners. Nonetheless, creative solutions, such as conducting interviews in local LGBTQ+ magazines, were beneficial. Although the study sample may seem small, it was sufficient for qualitative research to gain a deeper understanding of the participants’ experiences. Qualitative studies tend to have smaller sample sizes than quantitative ones; even a single case can be meaningful and provide valuable insight.^27^ The consensus is that the sample size can be manageable, typically ranging from 3 to 10 participants,^28^ or 6 to 10 to minimize the volume of data.^29^ Moreover, the study’s sample was homogeneous, comprising solely gay Christian men.

The sample was restricted to participants who could readily access and afford to travel to central London. Consequently, data from gay Christian men in social isolation living outside the capital were excluded. The participants who volunteered in the study were willing to engage in the workshops, which does not represent the full range of gay Christian men and potentially those who feel the most shame for their identity. It is essential to acknowledge the researcher’s prior involvement in this collaboration, as evidenced by the pilot study. The prior experience influenced both data interpretation and the framing of the study, though it provided the necessary resources to organize the collaborative element of the research with the Church.

### Conclusion and recommendations in practice

The nature of qualitative research is to open more questions about a given phenomenon. This research aimed to understand the experiences of gay Christian men attending a collaboration. The research offers an in-depth understanding of what it was like to attend a series of workshops, facilitated by a sexual health professional and the Church, at a specific time and location, with a sample of participants.

The influence of the collaboration on gay Christian men occurred on two levels: their self-perception and their relationships. These workshops facilitated personal growth, enabling some gay Christian men to reflect on themselves and learn from the presentations and resources provided by the co-facilitators. Conversely, others felt despondent about reconciling their faith with their sexuality. Participants from the most conservative denominations of Christianity experienced a profound sense of hopelessness linked to ongoing issues of shame and regret.

Participants who attended with a personal motivation to embrace the collaboration’s messages gained more than those who sought validation and acceptance. The environmental factors had a significant impact on the collaborative workshops. Some participants appreciated the physical layout and sense of community, while others emphasized the differences within the group.

The concept of collaboration emerged from my clinical caseload as a psychosexual therapist, where I recognized the importance of creating a space for gay religious men. Participants aligned their personal needs, such as acceptance (being gay and Christian), with the collaborative efforts. Maslow suggested that humans prioritize their needs based on motivation; when basic needs (such as food and shelter) are met, higher-level needs (such as love and belonging) can begin to motivate behavior. Those who attended the workshops were inspired to engage with the messages presented and derived significant benefits, as they had already fulfilled their fundamental needs, according to Maslow’s hierarchy of needs. In contrast, participants with an urgent need for validation and acceptance struggled to engage in the workshops fully or to progress further along the needs hierarchy. These individuals felt weighed down by the shame of being both gay and Christian. A primary contribution of this study is an adapted version of Maslow’s hierarchy of needs that highlights that gay Christian men can be free from shame and genuinely experience belonging and love only when they feel secure in their environment.

The adapted version of Maslow’s hierarchy serves as a new proposed model that could help understand the integration of sexuality and religion for gay Christian men and could potentially help anyone who identifies as a religious LGBTQI+. It highlights the need to address feelings of shame before high levels of Maslow’s needs can be met for this subset of the population.

The key recommendations from this study’s findings for sexual health or therapy professionals include evaluating human needs using an updated version of Maslow’s hierarchy. It would also be beneficial to refer individuals for personalized counselling. Practitioners could consider incorporating questions about religion and spirituality into patients’ sexual history assessments. Providing contemporary faith resources to support sexual minorities would be advantageous. Collaborating with local religious organizations or faith leaders is similarly advised. Furthermore, practitioners should seek additional training regarding the role of religion in individuals’ lives.

Furthermore, faith leaders and religious institutions could benefit from establishing targeted collaborations based on a contemporary interpretation of Maslow’s hierarchy of needs. This approach would foster inclusive environments for LGBTQ+ individuals within religious communities. Ultimately, this study suggests that religious sexual minorities require a welcoming atmosphere to feel included. Faith leaders could enhance their understanding of the needs of both religious and sexual minorities and consider partnering with a local sexual health clinic. Engaging in training or observing inclusive and LGBTQ+-affirming churches would also be beneficial.

Future research, with sufficient funding, can build upon the findings of this study. Sexual behaviour, “free from shame,” more generally, not just for gay Christian men, should be explored in relation to the hierarchy of needs. A larger sample of participants could allow a mixed-methodology approach, combining qualitative and quantitative methods, to be utilized. Quantitative measures could include validated questionnaires such as the Patient Health Questionnaire-9, Generalized Anxiety Disorder-7, Spiritual Wellbeing Questionnaire, Warwick–Edinburgh Mental Well-being Scale, International Index of Erectile Function, Satisfaction with Life Scale, Self-Compassion Scale, and Quality of Life Questionnaire. It would be helpful to use those scales before and after conducting another series of collaborations to measure their impact.

## Supplementary Material

Thematic_Analysis_Supplement_qfaf103

Supplement_two_demographics_qfaf103
