# Supplementary material for: The experience of gay Christian men attending collaborative workshops facilitated by a sexual health professional and a priest
Source: Sex Med. 2025 Dec 17;13(6):qfaf103. doi: 10.1093/sexmed/qfaf103 (PMC12710469; doi:10.1093/sexmed/qfaf103)
Supplement: Thematic_Analysis_Supplement_qfaf103 [file thematic_analysis_supplement_qfaf103.docx]

| Thematic Analysis | 15- Check points | Current research |
| --- | --- | --- |
| Transcription | 1. The data have been transcribed to an appropriate level of detail, and the transcripts have been checked against the tapes for ‘accuracy’. | The recordings (including images and interviews) were listened and transcribed using software called Google Docs. I typed the haiku poetry from handwritten forms. Once transcribed, I listened to the recordings again for accuracy. |
| Coding | 1. Each data item has been given equal attention in the coding process. | I used a software named NVivo to support the coding process. |
|  | 1. Themes have not been generated from a few vivid examples (an anecdotal approach), but instead the coding process has been thorough, inclusive and comprehensive. | I have made clear links to my interpretation of the data and how themes developed from a consistent coding process. |
|  | 1. All relevant extracts for each theme have been collated. | Each theme is linked to relevant extracts from data. |
|  | 1. Themes have been checked against each other and back to the original data set. | Codes were then grouped to sub-themes and subsequent themes, which were checked back and forward to original data. |
|  | 1. Themes are internally coherent, consistent, and distinctive. | I demonstrated the theme development of each method and later the overarching themes across data. |
| Analysis | 1. Data have been analysed – interpreted, made sense of – rather than just paraphrased or described. | Due to the large volume of data to analyse, I paid attention to the interpretation of the analysis by giving myself sufficient time to reflect and make sense of findings. |
|  | 1. Analysis and data match each other – the extracts illustrate the analytic claims. | I provided direct quotes from participants to demonstrate my analytic process. |
|  | 1. Analysis tells a convincing and well-organised story about the data and topic. | The analysis is structured around the data and with the main aim of answering the research question. |
|  | 1. A good balance between analytic narrative and illustrative extracts is provided. | I ensured that the analysis demonstrates sufficient vivid quotes from the participants either in text or images to demonstrate the narrative of the analytic story. |
| Overall | 1. Enough time has been allocated to complete all phases of the analysis adequately, without rushing a phase or giving it a once-over-lightly. | The analysis was a developing process that was not rushed in any way. In fact, once complete, I allowed myself to go back and reflect on the analysis, with fresh eyes. |
| Written report | 1. The assumptions about, and specific approach to, thematic analysis is clearly explicated. | I have explained the rationale of using thematic analysis. I appreciated the flexibility of thematic analysis whilst remaining consistent with the phenomenological thinking around understanding the experience of the participants. |
|  | 1. There is a good fit between what you claim you do, and what you show you have done. | I made every effort to be transparent between what I planned to do with my analysis and how I delivered it. |
|  | 1. The language and concepts used in the report are consistent with the epistemological position of the analysis. | The analysis maintained a phenomenological framework. I aimed to understand the experience of the participants, which was socially constructed within the collaborative workshops. |
|  | 1. The researcher is positioned as active in the research process; themes do not just ‘emerge’. | I aimed to be transparent with my reflexivity as a researcher and my active involvement in all aspects of the analytic process. |
