# Supplementary material for: The experience of gay Christian men attending collaborative workshops facilitated by a sexual health professional and a priest
Source: Sex Med. 2025 Dec 17;13(6):qfaf103. doi: 10.1093/sexmed/qfaf103 (PMC12710469; doi:10.1093/sexmed/qfaf103)
Supplement: Supplement_two_demographics_qfaf103 [file supplement_two_demographics_qfaf103.docx]

| Participant | Age | Ethnicity | Denomination | Relationship |
| --- | --- | --- | --- | --- |
| P010 | 67 | White British | Church of England | In a relationship |
| P011 | 45 | White British | Church of England | Single |
| P012 | 63 | White Other | Catholic | Single |
| P013 | 43 | Chinese | Church of England | Single |
| P014 | 28 | White British | Church of England | Single |
| P015 | 73 | White British | Church of England | In a relationship |
| P016 | 55 | White Irish | Catholic | Single |
| P017 | 73 | White British | Church of England | Single |
| P018 | 28 | Black British African | Charismatic | In a relationship |
| P019 | 27 | Black British African | Baptist/Pentecostal | Single |
| P020 | 40 | Any other Asian | Catholic | Single |
